# Enhanced myco-synthesis of selenium and zinc oxide nanoparticles and evaluating their anticancer activities and role against antibiotic resistance genes in certain bacterial strains

**DOI:** 10.1186/s12934-025-02795-w

**Published:** 2025-10-04

**Authors:** Amira Mohamed, El-Sayed R. El-Sayed, Zainab Zakaria, Mona H. Mohamed, Heba K. A. Elhakim

**Affiliations:** 1 Biotechnology Division, Faculty of Science, Cairo University, Giza, Egypt; 2 Plant Research Department, Nuclear Research Center, Egyptian Atomic Energy Authority, Cairo, Egypt; 3 Research and Development Department, Faculty of Pharmacy, Heliopolis University, Cairo, Egypt; 4 Faculty of Public Health, Saxony Egypt University, Cairo, Egypt; 5 Biochemistry Division, Faculty of Science, Cairo University, Giza, Egypt

**Keywords:** Selenium, Zinc, Nanoparticles, Endophytic, Anticancer, Antibiotic Resistance Genes

## Abstract

**Background:**

In an array to check microbial resistance against generally used antibiotics, it is essential to create innovative and efficient antimicrobial agents. Therefore, nanoparticles (NPs) with their antimicrobial activities describe an effective solution. In this study, we synthesized Selenium nanoparticles (Se-NPs) and zinc oxide nanoparticles (ZnO-NPs) using *Alternaria alternata* fungus, then their characterization were evaluated using several techniques.

**Results:**

We explored the potential of antimicrobial impact of Se-NPs and ZnO-NPs against negative and positive grams antibiotic resistance bacterial strains in combination with penicillin, Ceftriaxone and Cefipime. Moreover, antibiotic resistance gene expression was assessed after those treatments. The results demonstrated that Se-NPs and ZnO-NPs displayed antibacterial properties, while the expression of antibiotic resistance genes decreased when exposed to a combination of NPs and antibiotics. This suggests the presence of both synergistic and additive effects in these treatments. Furthermore, the cytotoxic effects of Se-NPs and ZnO-NPs were assessed, revealing their potent anticancer properties against MCF-7, A549, and HepG2 cancer cells and lower cytotoxic values for HFB-4 standard cell line. Ultimately, the production efficiency of both NPs was enhanced through gamma irradiation.

**Conclusions:**

According to the results, it seems that the green synthesis of Se-NPs and ZnO-NPs promotes environmental sustainability and cost-effective approach. This study provides insights into the development of new antibacterial and anticancer agents . The eco-friendly production of nanoparticles suggests also a sustainable approach to combating bacteria resistant to antibiotics.

## Introduction

With the advancements and utilization of nanotechnology over the past ten years, both metal and non-metal nanoparticles (NPs) have attracted increased interest because of their distinct properties in contrast to their bulk counterparts. The unique characteristics of NPs are entirely reliant on their morphology, dimensions, and the distribution range of the resulting particles [1]. Various approaches, including chemical, physical, and eco-friendly methods, have been employed for nanoparticles (NPs) synthesis. Among these, green synthesis is widely regarded as a cost-effective, environmentally sustainable, and streamlined technique, as it relies on natural biological reducing agents and stabilizers in NPs' formation [2, 3]. Mycogenic synthesis of NPs is a green biogenic process preferable to other druthers. Fungi are widely utilized in the biosynthesis of NPs due to their highly efficient fungal metabolites, which hold a vital significance in the formation of various NPs [4, 5]. Fungi are regarded as a valuable recent addition to the list of microorganisms utilized in the synthesis of NPs. The rise of antibiotic resistance has become a growing concern, as bacteria continue to evolve and develop adaptive defenses against traditional antibiotics at a rapid pace [6]. Due to the growing demand for novel antimicrobial substances, NPs have been suggested for infection treatment, as they eliminate bacteria through mechanisms distinct from those of traditional antibiotics [7]. Because of their comparatively low toxicity in human cells, nanomaterials are frequently regarded as a potential substitute for antibiotics in controlling bacterial infections [8]. Zinc and selenium play essential roles as vital nutrients in living organisms [9, 10]. It has been designated that ZnO-NPs as a superb prospective in biological treatments, because of the antimicrobial potential and also an alluring choice since they are safe to the human body and environment at least concentrations [11–14]. Furthermore, various research studies have documented the effectiveness of ZnO-NPs in suppressing the growth of a wide range of pathogens [13–15] Zinc is a vital trace element involved in numerous physiological processes within the body. Additionally, it possesses the capability to function as a substitute for traditional antibiotics [9, 10, 15].

Selenium (Se) has unique properties and great potential in several fields like physics, chemistry, and biology [16]. It is a crucial component for humans, animals, and microorganisms, contributing significantly to the production of selenoenzymes through biosynthesis [16]. Many activities and properties make Se-NPs a perfect choice for various medical and industrial uses [17]. Se-NPs exhibit diverse biological and medicinal properties, including antimicrobial, antioxidative, and anticancer effects [18]. Additionally, they hold significant value in optical and electronic applications [17].

Currently, a major global concern is the resistance of various drugs to antimicrobial treatments [18]. Successful and expensive therapy is required to avoid encouraging incapacities or indeed passing for the most antibiotic-resistant diseases [19, 20]. Besides, current considerations demonstrated that tumors might end up safe to chemotherapy, much like bacterial resistance against conventional pharmaceuticals [21]. On the other hand, the current cancer medications have detailed various side impacts on patients’ wellbeing. In this manner, we are in frantic requirement of a quick cure distinctive from the obsolete and customary ones.Here, we describe the synthesis of Se-NPs and ZnO-NPs  utilizing an endophytic fungus. The antimicrobic activity of Se-NPs and ZnO-NPs was evaluated against gram positive and negative antibiotic resistance bacterial strains. The effect of those  NPs  was confirmed through the assessment of gene expression related to antibiotic resistance using the current bacterial strains. Additionally, the anti-cancer potential of these NPs was examined opposed to cancer and normal cell lines. Finally, research was studied on how gamma irradiation affected the synthesis of both NPs.

## Materials and methods

### Plant samples collection and isolation of endophytic fungi

Samples of different plant species were gathered from multiple regions across Egypt. Plant specimens were meticulously extracted using a sanitized, precision blade and then transported to the laboratory. The specimens of these plant were utilized to isolate endophytic fungi following the method outlined in earlier research [22]. In summary, the plant specimens were broken into smaller pieces and underwent surface disinfection, which included treatment with 70% ethanol, followed by exposure to 0.1% mercuric chloride (HgCl_2_). The obtained pieces were allowed to air-dry on a sterile, dry filter paper before being evenly distributed on potato dextrose agar supplemented with streptomycin and tetracycline. Subsequently, the Petri dishes were placed in incubation at 25 °C in darkness and observed daily.

### Screening of isolated fungal endophytes for the biosynthesis of Se-NPs and ZnO-NPs

#### Fermentation and preparation of fungal extract

Sugarcane bagasse was obtained from a local mill (Menia El-Kamah, Egypt). The collected bagasse samples were dehydrated in an oven at 50 °C and subsequently ground to achieve a grain dimension varying between 0.2 and 0.5 mm. Spores from five-day-old fungal cultures were collected, and their concentration was regulated to a 10^6^ spores ml^−1^ with the aid of a hemocytometer. Each ten-gram portion of sugarcane bagasse was separately placed into 250 mL containers. A 25 mL volume of mineral salt solution was added to Erlenmeyer flasks, serving as a moisturizing agent. The solution contained (g L^−1^): 0.01 of FeSO_4_·7H_2_O, 0.05 of MgSO_4_·7H_2_O, and 0.05 of KCl. The containers underwent sterilization in an autoclave set to 121 °C for a duration of 20 min. Once they had cool down to an ambience temperature, 1 ml aliquot of the spore suspension was added. The flask insides were then mixed thoroughly before being retained in an incubator at 30 °C for a duration of 10 days. Later, during the incubation period, the inoculated flasks were retrieved and subjected to extraction with absolute ethanol for one hour on a rotational shaker at an ambient temperature and passed within filter paper of Whatman No.1 for filtration. The extract  underwent additional centrifugation at 10,000 rpm for 10 min, and the obtained sample was utilized for NPs  synthesis.

#### Synthesis of NPs

Sodium selenite (Na_2_SeO_3_) and zinc sulfate (ZnSO_4_·7H_2_O) were purchased from Sigma Aldrich (St. Louis, MO). For Se-NPS synthesis, 100 ml of fermented sugarcane bagasse extract was placed in an Erlenmeyer flask and combined with an equal volume of sodium selenite solution (1 mM final concentration). For ZnO-NPs synthesis, 100 ml of fermented bagasse extract was placed in an Erlenmeyer flask and combined with an equal volume of zinc sulfate solution (2 mM final concentration). The mixtures were continuously agitated at room temperature for six hours. A distinct color shift or precipitate formation in the final mixture was considered a reliable sign of metal salt reduction. Under the same conditions, negative controls contained only salt solutions, while positive controls included extracts from each fungal isolate, were tested simultaneously.

### Fungal strain

*Alternaria alternata* AUMC15177, isolated from *Citrus medica* bark, exhibited the ability to reduce both metal salts. The fungus, cataloged as AUMC15177, was stored at the Mycological Centre of Assiut University (AUMC) in Assiut, Egypt [https://www.aun.edu.eg/sp_units/node/42688].

### Identification of the positive fungus

The colony’s fungal and cultural characteristics were analyzed in detail using Czapek’s yeast autolysate agar (CYA) medium. The following components are present in this medium per litre: 5.0 g of yeast-derived extract, 3.0 g NaNO_3_, 0.5 g MgSO_4_·7H_2_O, 0.5 g KH_2_PO_4_, 0.5 g KCl, 0.01 g FeSO_4_·7H_2_O, and 3.0 g of sucrose [23]. The fungus identity was affirmed by molecular characterization utilizing PCR enhancement of the ITS1-5.8S-ITS2 rRNA [24]. Solgent, based in Daejeon, South Korea, conducted the isolation and sequencing of the genomic DNA. The fungal sequences were submitted to GenBank for an accession number. The analysis was conducted utilizing BioEdit version 7.0.1 along with the BLAST tool, accessible via http://www.ncbi.nlm.nih.gov/. The MEGA 6.0 program was utilized to construct both maximum-likelihood and neighbor-joining phylogenetic tree.

### Separation, purification, and characterization of Se-NPs and ZnO-NPs

Prior to analysis, the reaction mixtures were isolated, and the produced NPs were refined. Following several cycles of spinning at 12,000 rpm for 20 min each, the process of centrifugation was completed, the two NPs were collected separately, dried at 50 °C after being rinsed with ethanol and sterile deionized water. The Se-NPs and ZnO-NPs powders were separately dispersed in HPLC-grade ethanol, subjected to ultrasonic processing, and subsequently analyzed using the following techniques:

1. **Fourier Transform Infrared Spectroscopy (FT-IR):** FT-IR spectra were attained within the 400–4000 cm^−1^ range utilizing an IRAffinity-1 spectrophotometer (Shimadzu, Japan).
2. **X-ray Diffraction (XRD):** The crystallographic structure was examined via XRD and Cu-Kα radiation (λ = 1.5406 Å) at 40 kV and 40 mA, covering the angular range of 10° ≤ 2θ ≤ 80° with a BRUKER diffractometer (D8 DISCOVER with DAVINCI design, USA).
3. **Transmission Electron Microscopy (TEM):** The morphologic characteristics of the synthesized nanoparticles (NPs) analyzed utilizing a High-Resolution Transmission Electron Microscope (JOEL 2100 model) operated at a hurrying voltage of 8000 kV, focusing on nanoparticle imaging.
4. **Zeta Potential Analysis:** Using a Zetasizer Nano ZS (Malvern Instruments, Worcestershire, UK) and Dynamic Light Scattering (DLS), particle size scattering and stability were evaluated.

### Cytotoxicity assay

This research utilized four distinct cell line types: breast carcinoma cells (MCF-7), normal human melanocytes (HFB-4), pulmonary epithelial cancer cells (A549), and hepatic cancer cells (HePG-2). These cell lines were kept in Dulbecco’s Modified Eagle Medium (DMEM) after being acquired from the NAWAH Scientific Centre, Cairo, Egypt, supplemented with a 10% concentration of fetal bovine serum, along with 100 µg of streptomycin and 100 units of penicillin. The controlled environment in which the cell cultures were kept had a constant temperature of 37 °C, within a moisture-rich atmosphere composed of 5% CO_2_ and 95% O_2_. Serial dilutions of Se-NPs and ZnO-NPs were formulated by dispersing them in fertile phosphate-buffered saline (PBS) within a concentration range of 3.906–500 µg/ml. The cytotoxic effects of these NPs were assessed using the MTT prove (3-(4,5-dimethyl-2-thiazolyl)-2,5-diphenyl-2H-tetrazolium bromide) [25]. An aggregate of 10,000 cells (10^4^ per well) were plated as monolayers in a 96-well tissue culture plate, except for three wells designated as controls. The cells were kept in a humid environment with 5% CO_2_ at 37 °C for the entire night. Following incubation, sequential dilutions of Se-NPs and ZnO-NPs were introduced to the cultures. The cells were incubated for an additional 48 h before being rinsed with PBS. Subsequently, 50 µl of the MTT component was introduced into each well. Following a 4-h incubation, 50 µl of 0.5 µg/ml of DMSO was introduced for the purple formazan crystals to dissolve generated through MTT reduction. The absorbance at 750 nm was measured using a BioTek microplate ELISA reader (USA). Each concentration of both control and test samples was evaluated in triplicate, and the entire experiment was conducted three times. The viability rate of cells was assessed for every treatment condition.

### Antibacterial activity of Se-NPs and ZnO-NPs

Four strains of bacteria resistive to antibiotics were utilized (Table 2). Three antibiotics were employed: Ceftriaxone at a concentration of 1.0 g (Rameceftraxe, produced by RAMEDA Pharmaceuticals), Cefipime 1.0 g (Board Spectrum Antibiotic, PHARCO B Company), and Benzathine penicillin at a dosage of 1.2 M.I.U. (Depo-Pen, manufactured by Medical Union Pharmaceuticals Company). To assess the minimum inhibitory concentrations (MIC), a standardized bacterial count was protected overnight with Se-NPs and ZnO-NPs in a 96-well plate, sequential dilutions of antibiotics, with each condition tested in triplicate [26]. Using a general-purpose medium, the bacterial strains were initially cultivated on an agar plate and brooded for 18–24 h. Subsequently, 3–5 distinct colonies from each strain were transferred into tubes including 4.0–5.0 ml of Nutrient Broth. These cultures were subsequently incubated at 37 °C until their turbidity measured 0.25 at 630 nm. To standardize the bacterial strain concentration, the turbidity of the aggressively proliferating broth culture was modified by adding additional broth, achieving an estimated density of 2 × 10^6^ CFU/ml per well. Following this, antibiotics, Se-NPs, and ZnO-NPs were introduced in a two-fold serial dilution sequence. The plate was subsequently left to incubate at 37 °C for a full day. The Minimum Inhibitory Concentration (MIC) refers to the smallest dosage of antibiotics or NPs required to entirely suppress bacterial proliferation.

### Antibacterial action of the combinations of Se-NPs and ZnO-NPs and antibiotics

The checkerboard assay was employed to identify the cooperative effects of combining Se-NPs and ZnO-NPs with antibiotics, including Benzathine penicillin, Ceftriaxone, and Cefipime [27]. Serial dilutions of both antibiotics and nanoparticles were employed to assess possible synergistic effects between them. To determine the Fractional Inhibitory Concentration (FIC), a two-fold serial dilution was carried out for Benzathine penicillin, Ceftriaxone, and Cefepime, with concentration levels spanning from 5 to 0.039 mg/ml. Similarly, Se-NPs and ZnO-NPs were diluted in a series, with concentrations varying between 1 and 0.031 mg/ml. The antibiotic levels were gradually reduced in a vertical manner and paired with decreasing concentrations of Se-NPs and ZnO-NPs to assess the Fractional Inhibitory Concentration (FIC). Negative control that consisted solely of broth was used. Additionally, a growth control was included, which consisted of the bacterial strain culture lacking any antibacterial agents. In individual plates, wells were filled with 100 µl of bacterial cultures at a density of 2 × 10^6^ CFU/ml, excluding the negative control. Following this, the plates were hatched at 37 °C for an entire night. The designated equation was used to calculate the Fractional Inhibitory Concentration (FIC);

$$\mathbf{FIC}=[\mathbf{C}_\mathbf{NP}/\mathbf{MIC}_\mathbf{NP}]+[\mathbf{C}_\mathbf{Ab}/\mathbf{MIC}_\mathbf{Ab}]$$

Where C_NP_ represents the NPs concentration, and C_Ab_ denotes the antibiotic concentration in the mixture. MIC_NP_ refers to the minimum inhibitory concentration (MIC) of the synthesized NPs, while MIC_Ab_ corresponds to the MIC of the antibiotic. To analyze the outcomes: Synergistic effect (FIC index < 0.5), Additive effect (FIC index 0.5–2), Indifferent effect (FIC index 2–<4), and Antagonistic result (FIC index ≥ 4). These analyses conform to the guidelines and suggestions set by EUCAST [28, 29].

### Assessment of antibiotic resistance gene activity

The gene expression levels related to antibiotic resistance were analyzed by comparing bacterial treatments using each NP combined with a specific antibiotic compared to those treated solely with the antibiotic. This comparison was conducted for a Gram-negative strain (*E. coli* DSMZ5923) and a Gram-positive strain (MRSA DSMZ28766) in the experiment. A set number of bacteria (2 × 10^6^ CFU/test per strain) was grown with sub-MIC levels of antibiotics, with or without NPs. The cultures were left to incubate overnight. Subsequently, the bacterial specimens underwent centrifugation at 12,000 rpm for one minute, followed by the careful removal of the supernatant. The bacterial cell-containing pellet was preserved at −80 °C until it was needed for subsequent RNA isolation. The extraction of total RNA from bacterial strains was conducted utilizing the Easy-Spin™ [DNA-free] Total RNA Extraction Kit (iNtRON BIOTECHNOLOGY). To remove genomic DNA, DNase treatment was applied using a Thermo Scientific kit (USA) specifically designed for this procedure. The isolated RNA was subsequently converted into complementary DNA (cDNA) through reverse transcription, utilizing 150 ng of template RNA along with the Maxime RT PreMix Kit. A real-time PCR system (Thermo Scientific Kit, USA) was employed to quantify variations in antibiotic resistance gene expression among bacterial strains. During the PCR process, a 1 μl sample of template cDNA was utilized, accompanied by 1 μM of both the forward and reverse primers (Table 3). Moreover, a volume of 12.5 μl of Maxima SYBR Green qPCR Master Mix (2×) was incorporated. The heat phase was executed using a three-phase procedure that included a total of 40 repetitions. This procedure involved denaturing the strand at 95 °C for 15 s, allowing primer binding at 60 °C for 30 s, and extending at 72 °C for another 30 s. The real-time PCR analysis was performed via the MxPro3005 system from Agilent Technologies. The variation in antibiotic resistance gene expression was assessed by comparing samples exposed solely to antibiotics with those subjected to both NPs and antibiotics. This computation relied on a specific formula and was standardized using the 16S rRNA gene as a benchmark. The primers employed in our study were selected based on a previous publication [30].

**Table 1:** Calculated size, mean size, size distribution, zeta potential values, and polydispersity index (PDI) of the myco-synthesized NPs

Studied parameters	Se-NPs	ZnO-NPs
Calculated size (nm)	45.83	61.77
Size distribution (nm)	25–86	32–91
Mean size (nm)	51.96	65.43
Zeta potential (mV)	−21.89	−20.74
PDI	0.107	0.116

Particle size was calculated from the Scherrer equation. Mean size, size distribution, zeta potential, and PDI were obtained from the DLS analysis as described in Materials and Methods

### Effect of gamma irradiation on synthesis efficiency of Se-NPs and ZnO-NPs

The efficacy of extract of the fermented sugarcane bagasse as induced by gamma irradiation on the fabrication of Se-NPs and ZnO-NPs was investigated. The spores of *Alternaria alternata* AUMC15177 were disrupted following the previously outlined procedures in this study, then placed in vials confirmed with paraffin and exposed to gamma radiation at intensities of 500, 1000, 2000, 4000, and 8000 Gy. To prevent photo-reactivation, the spore suspensions exposed to gamma radiation were stored in complete darkness at 4 °C for the duration of the night. After that, flasks were inoculated, incubated, extracted and the extract was used for the  synthesis of Se-NPs and ZnO-NPs, as described earlier. The efficiency of Se-NPs and ZnO-NPs synthesis was estimated as stated by this formula:

$$\text{Synthesis efficiency}(\%)=[(\text{C}_{\rm o}-\text{C})/\text{C}_{\rm o})] \times 100$$

where* C* represents the weight of the synthesized Se-NPs and ZnO-NPs*,* and* C*_o_ is the concentration of Se and Zn in the precursor solution.

### Statistical analysis

The tentative findings were represented using the average value and standard deviation. The calculated average reflects data obtained from three independent experiments, with each measurement taken in triplicate. A One-Way ANOVA was performed to determine statistical significance, followed by the Least Significant Difference (LSD) test, with a predefined significance threshold of 0.05. The data analysis was executed using SPSS software, version 22 (IBM, New York).

## Results and discussion

### Screening of endophytic fungi for the myco-synthesis of Se-NPs and ZnO-NPs

Several fungal endophytes were isolated, and all the isolated fungi were cultivated on sugarcane bagasse, and their capability to reduce the salts to of nanoparticles of Se and ZnO was assessed (Data not shown). The screening results revealed that the isolated number SRZ2020, isolated from *Citrus medica* bark, demonstrated the ability to decrease both kinds of salts. This was clearly demonstrated by noticeable shifts in color from colorless to red in case of Se-NPs and formation of white precipitate in the case of ZnO-NPs. As a result, this specific fungal strain was selected for further detailed analysis and the application of subsequent experimental techniques. Generally, fungal endophytes are gaining high importance in the field of nanotechnology as this class of fungi exhibit increased metabolic activity in comparison to their free counterparts [31].

**Fig. 1:**
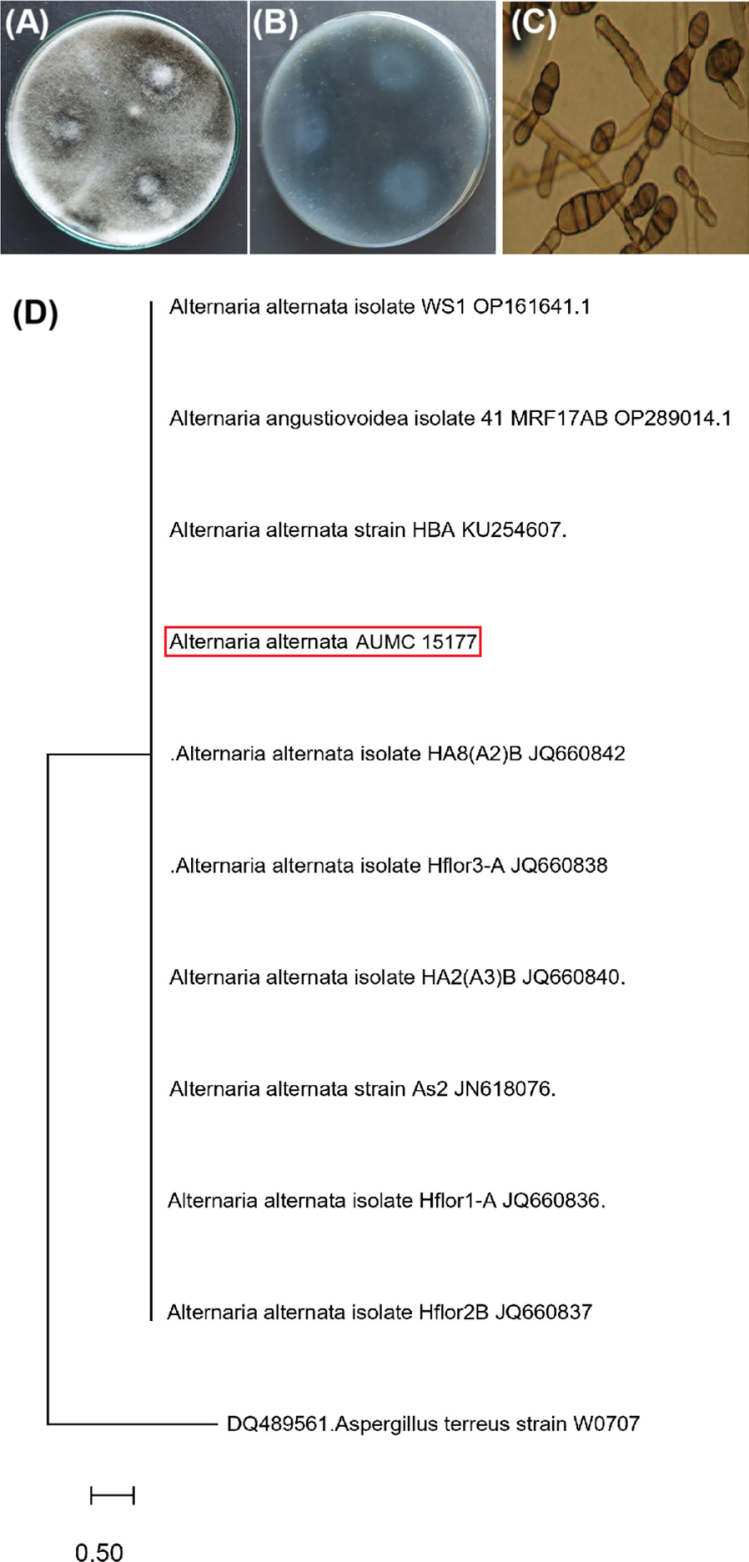
Morphological and molecular characteristics of the fungus used in preparation of Se-NPs and ZnO-NPs. Front view of the growth after incubation for 10 days at 30 °C **(A).** Reverse view of the growth after incubation for 10 days at 30 °C **(B)**. Microscopic features of growth showing conidiophore and conidial heads **(C)**. Phylogenetic tree of the *Alternaria alternata* AUMC15177 and other closely related strains** (D)**

The SRZ2020 isolate was identified by morphological and molecular methods. The fungal strain’s colony characteristics, as observed on the CYA medium, is shown in Fig. 1A–B. The front (Fig. 1A) and reverse (Fig. 1B) views of the fungal colony displayed dark pigmentation after 10 days of incubation at 25 °C. In contrast, the microscopic observation revealed elongated chains of conidia, each characterized by a peak and both transverse and longitudinal septation (Fig. 1C). The fungus was fully identified through molecular characterization. The fungal strain’s 18S rRNA was amplified and subsequently sequenced. The obtained sequence was submitted to GenBank with the accession number OR119850 and subsequently aligned with similar sequences through BLAST tools. A phylogenetic tree was generated based on the alignments of the ITS1-5.8SITS2 region. As depicted in Fig. 1D, the tree revealed a perfect 100% identity with *A. alternata* strains.

### Synthesis and characterization of Se-NPs and ZnO-NPs

All the isolated fungal endophytes were cultivated on sugarcane bagasse through solid-state fermentation, then extracted. The ability of the prepared extracts was tested to separately reduce sodium Selenite and Zinc Sulphate. After a 6 h of mixing, the myco-reductions were noticed where the colorless mixtures turned to deep red color (for Se-NPs) and white precipitate (for ZnO-NPs), representing the formation of the NPs. Our observations agreed with prior reports relating the fungal synthesis of Se-NPs [33] and ZnO-NPs [32].

**Fig. 2:**
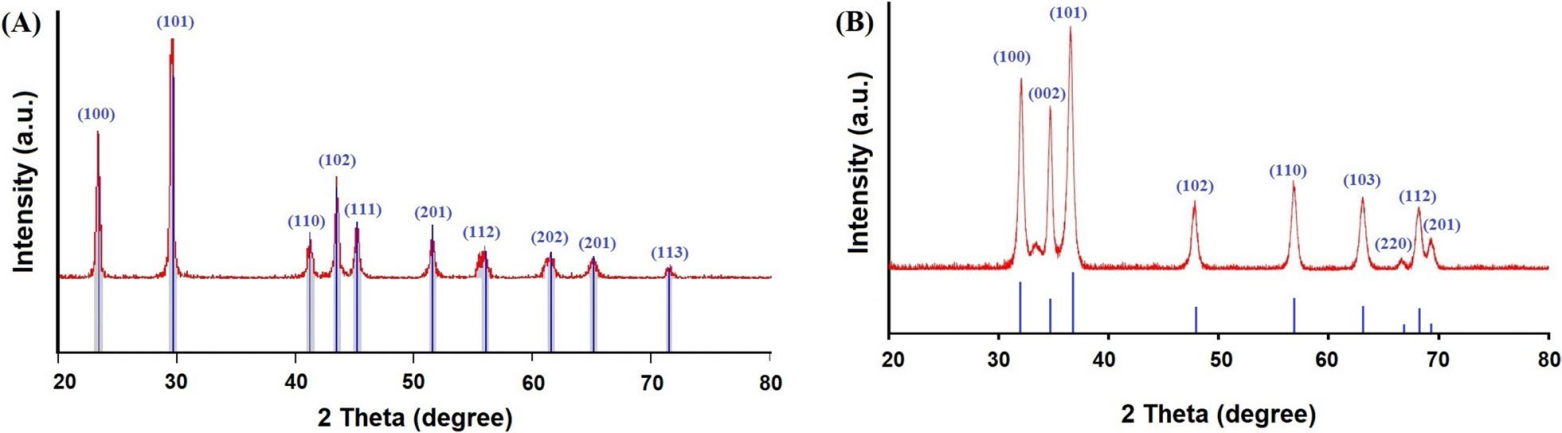
X-ray diffraction patterns of Se-NPs **(A)** and ZnO-NPs **(B)** myco-synthesized by *Alternaria alternata* AUMC15177 fermented sugarcane bagasse extract

**Fig. 3:**
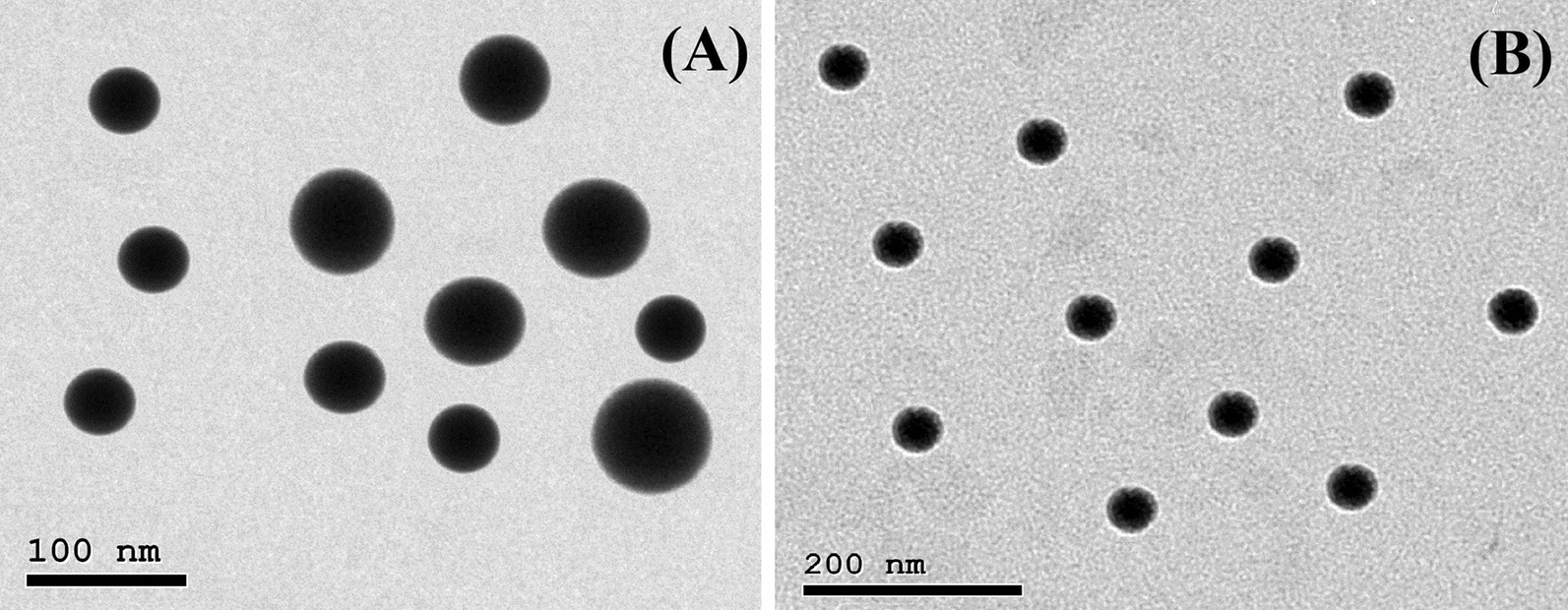
High Resolution TEM micrographs of Se-NPs **(A)** and ZnO-NPs **(B)** myco-synthesized by *Alternaria alternata* AUMC15177 fermented sugarcane bagasse extract

The myco-synthesized NPs in this study were characterized by XRD. Figure 2 shows the recorded XRD patterns of the Se-NPs (Fig. 2A) and ZnO-NPs (Fig. 2B) synthesized applying the extract of the fermented sugarcane bagasse from the *Alternaria alternata* AUMC15177. The XRD patterns of Se-NPs reveal diffraction peaks that validate their hexagonal crystalline structure, aligning with the specifications outlined in the Joint Committee on Powder Diffraction Standards (JCPDS) reference card No. 060326 [34]. Meanwhile, the diffraction peaks observed in the XRD analysis of ZnO-NPs verifies their hexagonal crystalline structure, aligning with the specifications outlined in the Joint Committee on Powder Diffraction Standards (JCPDS) reference card No. 361451 [35]. The data obtained in Table 1 denote that the calculated sizes of the produced Se-NPs and ZnO-NPs were 45.83 and 61.77 nm, respectively. According to DLS measurements (Table 1), particle size distributions were within the ranges of 32–91 nm (for ZnO-NPs) and 25 – 86 nm (for Se-NPs). Furthermore, the recorded data (Table 1) implies that the mean sizes of the respective NPs are 51.96 and 65.43 nm. The recorded zeta potential values are −21.89 and −20.74 mV, respectively. PDI for Se-NPs is 0.107 and 0.116 for ZnO-NPs (Table 1). The morphology of Se-NPs and ZnO-NPs synthesized using the extract of the fermented sugarcane bagasse from the *Alternaria alternata* AUMC15177 was studied by High Resolution transmission electron microscopy (HRTEM). Figure 3 illustrates TEM micrographs of Se-NPs (Fig. 3A) and ZnO-NPs (Fig. 3B) where spherical and monodispersed were observed. Previous reports used a variety fungal species such as *Trichoderma harzianum*, *Phoma glomerata*, *Aspergillus oryzae*, and *Monascus purpureus* to generate Se-NPs and ZnO-NPs with different shapes and sizes [36–38].

### Proposed mechanism of the myco-synthesis process

**Fig. 4:**
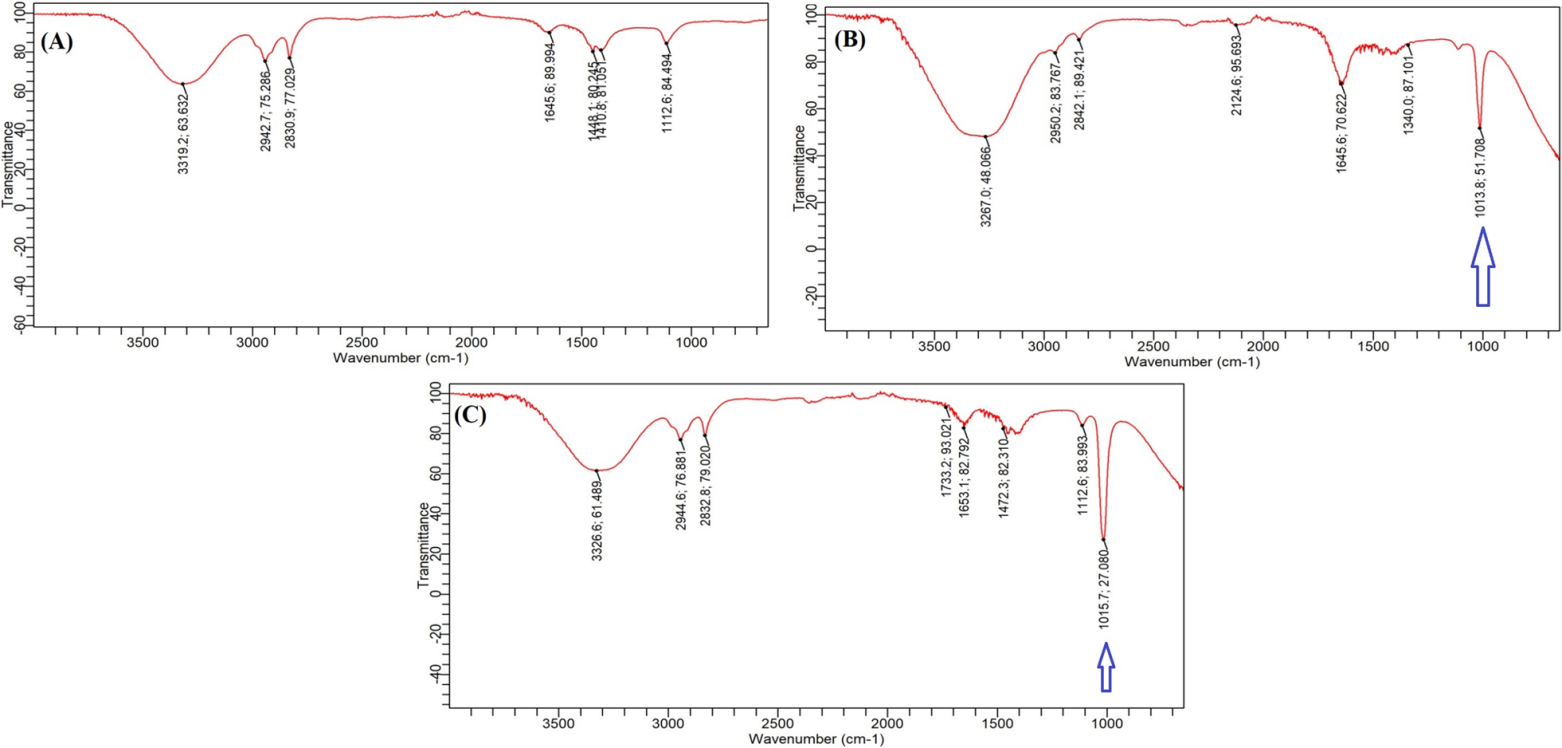
FTIR spectra of the *Alternaria alternata* AUMC15177 fermented sugarcane bagasse extract **(A)** and the myco-synthesized Se-NPs **(B)** and ZnO-NPs **(C)**

**Fig. 5:**
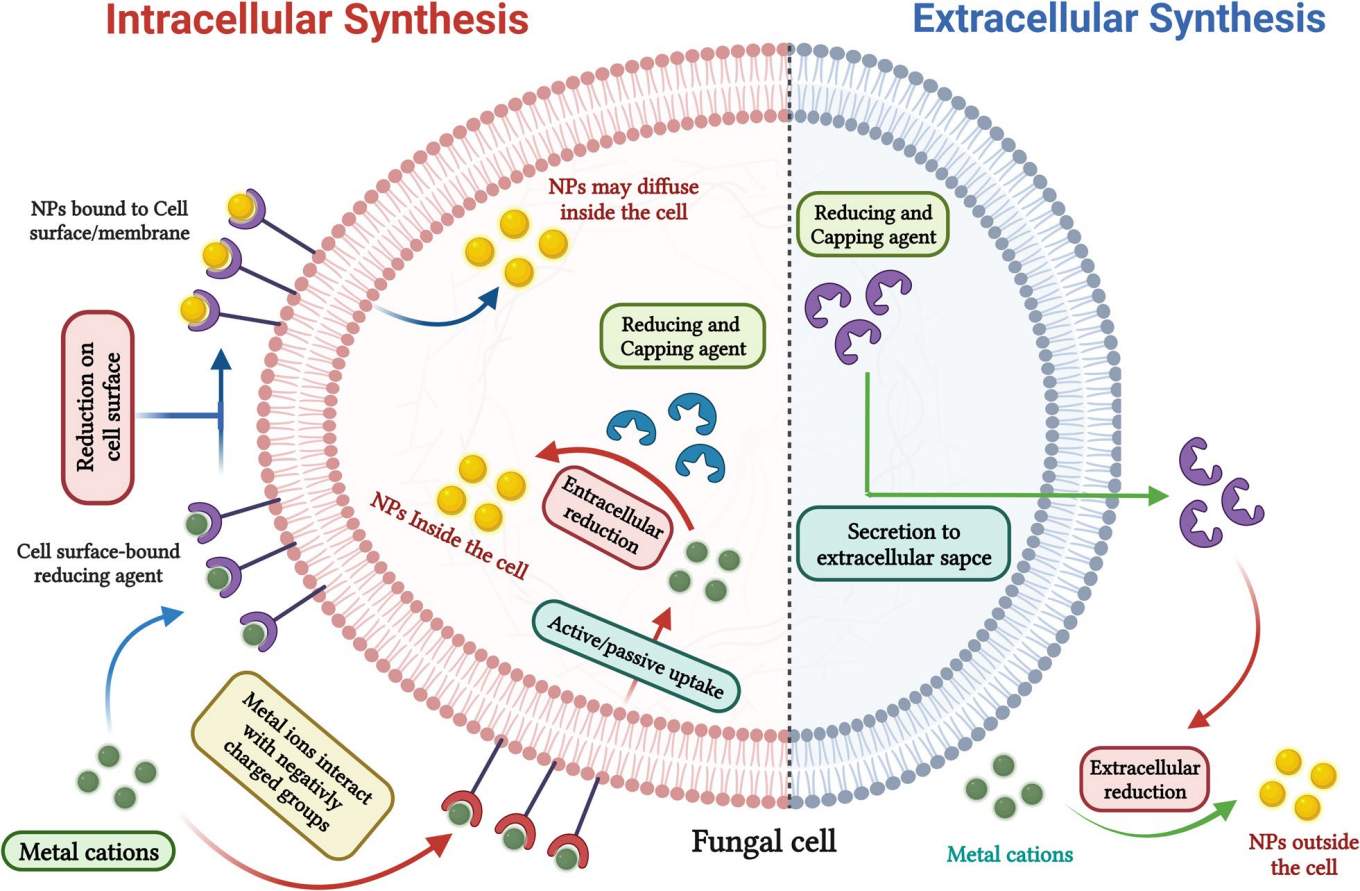
Proposed mechanism of Se-NPs and ZnO-NPs myco-synthesis by* Alternaria alternata* AUMC15177 fermented sugarcane bagasse extract

Currently, substantial advancements are being made in studies focusing on the biosynthesis of Se-NPs and ZnO-NPs utilizing various fungal species [32, 33]. In fact, fungi have demonstrated their effectiveness as optimal biotechnological tools for the eco-friendly synthesis of nanomaterials [39]. Moreover, the expense associated with producing nanomaterials through fungal synthesis can be substantially lowered by employing agro-industrial wastes as inexpensive substrates in solid-state fermentation. This approach not only minimizes production costs but also addresses issues related to environmental pollution and the safe disposal of waste materials [33]. In the current study, FTIR spectroscopy analyzed the interactions between myco-synthesized NPs and the extract of fermented sugarcane bagasse derived from *Alternaria alternata* AUMC15177. Presents the obtained FTIR spectra within the 400–4000 cm^−1^ range. Our findings indicated the emergence of extra bands at 1013 cm^−1^ (Se-NPs) and 1015 cm^−1^ (ZnO-NPs) in both spectra. Notably in Fig. 4, these bands do not match any of those detected in the extract of the fermented sugarcane bagasse from the *Alternaria alternata*. The collected data also reveal that both spectra exhibit key bands linked to primary amines, phenols, C–H stretching in CH_2_ units, and phenyl rings [32, 33]. Additionally, the spectra display COO^−^ groups, represented by two distinct bonds as C–O and C=O, also along with O–H bonds found in water [34–36]. This indicates that the extract derived from fermented sugarcane bagasse from the *Alternaria alternata* AUMC15177 in the myco-synthesis process. The results of this research are consistent with earlier studies that emphasized the significance of fungal cultures containing bioactive metabolites, extracellular enzymes, and proteins [32, 33] for the myco-synthesis of NPs and their subsequent stabilization [37, 38]. Various studies have suggested multiple mechanisms for the fungal-based synthesis of diverse NPs, involving both intracellular and extracellular activities of metabolites, proteins, enzymes, and other biomolecules. Figure 5 illustrates a potential pathway for the biosynthesis of NPs by fungi. Thus, we suggest that the myco-mediated synthesis of Se-NPs and ZnO-NPs in this research occurs through two separate stages: first, the biological reduction of metal ions, followed by the capping and stabilization of the generated NPs [32, 33, 39, 40].

### Cytotoxicity evaluation of Se-NPs and ZnO-NPs

**Fig. 6:**
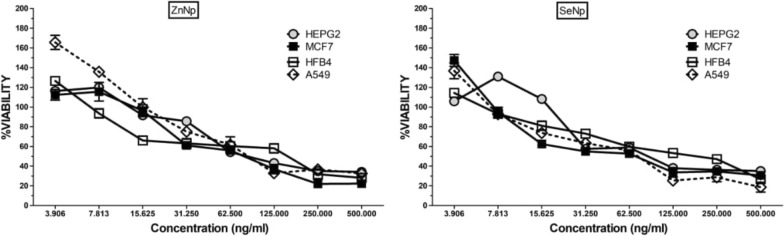
Cytotoxicity of the myco-synthesized Se-NPs **(A)** and ZnO-NPs **(B)** against normal human melanocytes (HFB-4), breast cancer cell lines (MCF-7), liver cancer cell lines (HePG-2), and lung epithelial cancer cell lines (A549)

This research utilized four distinct cell lines—HFB-4, MCF-7, HEPG-2, and A549. Both cancerous and non-cancerous cell lines were treated with progressively diluted concentrations of Se-NPs and ZnO-NPs to evaluate their cytotoxic effects on normal and malignant cells. As illustrated in Fig. 6A, the IC50 concentrations of Se-NPs for the HePG-2, MCF-7, HFB-4, and A549 cell lines were determined to be 90.5, 71.8, 188.2, and 76.8 µg/ml, respectively. The IC50 values of ZnO-NPs (Fig. 6B) were determined to be 83.5, 82.7, 164.2, and 88.4 µg/ml for the HePG-2, MCF-7, HFB-4, and A549 cell lines, respectively. The findings validated the anticancer potential of Se-NPs and ZnO-NPs against MCF-7, HepG2, and A549 cancer cells while demonstrating a lower cytotoxic effect on the HFB-4 normal cell line. Previous literature has recommended that Se-NPs and ZnO-NPs have cytotoxic effects on cancer cell lines (HeLa and MCF-7), highlighting its potential as a promising candidate for further exploration as a possible anticancer agent. The results demonstrate NPs are effective in inhibiting the growth and functionality of cancer cells while exhibiting lower toxicity toward healthy cells, making it a promising trait for anticancer agents. Too, additional study suggests evaluating the cyto-genotoxic effects of Se-NPs, especially in MCF-7 human breast cancer cells [33, 41]. Moreover, the in vitro anticancer results distinctly reveal that the newly developed Se-NPs exhibit significant potential in the field of therapeutics. Similarly, the nanoconjugates of selenium nanoparticles and doxorubicin facilitate the cellular uptake of the antimicrobial agent, thereby enhancing its retention and in this manner affecting its cytotoxic impacts against tumor cell [16, 42]. Studies have shown that biologically synthesized spherical Se-NPs, exhibit cytotoxic effects inhibiting cancer cell proliferation by approximately [16]. This crucial discovery provides insight into the potential genotoxic effects of Se-NPs and ZnO-NPs on cancer cell lines.

### Antibacterial action of ZnO-NPs, Se-NPs, and their combinations with antibiotics

The antimicrobial properties of Se-NPs and ZnO-NPs were assessed against multiple strains of pathogenic bacteria, encompassing both positive and negative gram types. The antimicrobial potency of these NPs  was subsequently evaluated in comparison to three distinct antibiotics. Our results demonstrates that Se-NPs and ZnO-NPs displayed broad antibacterial properties, surpassing the effectiveness of the administered antibiotics. The MIC test findings verified that all four bacterial strains exhibited resistance to Ceftriaxone, Benzathine penicillin, and Cefipime. The minimum inhibitory concentrations (MIC) of Se-NPs and ZnO-NPs for these strains were determined to be 0.125 and 0.5 mg/ml, respectively. In comparison, the MIC values for Ceftriaxone were recorded as 5 mg/ml for the MRSA#1 strain (L2-16ST347), 2.5 mg/ml for the MRSA#2 strain (L2-15ST13), and 5 mg/ml for both *E. coli* strains. Similarly, Benzathine penicillin demonstrated MIC values of 5 mg/ml for MRSA#1 (L2-16ST347), 2.5 mg/ml for MRSA#2 (L2-15ST13), and 5 mg/ml for the *E. coli* strains. Meanwhile, Cefepime exhibited MIC values of 2.5 mg/ml for MRSA#1 (L2-16ST347), 0.3125 mg/ml for MRSA#2 (L2-15ST13), and 5 mg/ml for both *E. coli* strains. The antibacterial properties of Se-NPs and ZnO-NPs align with earlier research, which has similarly validated their antimicrobial effects [41, 42]. Furthermore, the antibacterial possessions of the NPs could result from their ability to infiltrate and disrupt the bacterial cell wall and membrane, ultimately leading to cell death. Additionally, they may influence the expression of bacterial genes essential for growth [43]. BiosynthesizedSe-NPs exhibited potent suppression of growth and caused localized damage to pathogens. A greater release of proteins and polysaccharides occurred following interaction with NPs. Previous reports revealed structural damage to cell walls and membranes and the primary antibacterial mechanism of - was the generation of ROS [41, 42].

**Table 2:** MIC (mg/ml) and FIC Index of Se-NPs, ZnO-NPs, three antibiotics, and the combination between them

Bacterial strain	Combination	MIC		FIC	Effect
		NPs	Antibiotic		
MRSA#1 L2-16ST347	Ceftrixone + ZnO-NPs	0.25	1.25	0.75	Additive
	Cefipim + ZnO-NPs	0.03125	0.15625	0.325	Synergetic
	Penicillin + ZnO-NPs	0.25	1.25	0.75	Additive
	Se-NPs + Penicillin	0.125	2.5	1.5	Additive
	Se-NPs + Cefipim	0.03125	0.15625	0.3125	Synergetic
	Se-NPS + Ceftrixone	0.125	0.625	1.125	Additive
MRSA#2 L2-15ST13	Ceftrixone + ZnO-NPs	0.03125	0.15625	0.31	Synergetic
	Cefipim + ZnO-NPs	0.015625	0.078125	0.68	Additive
	Penicillin + ZnO-NPs	0.25	1.25	2.50	Indifferently
	Se-NPs + Penicillin	0.125	2.5	1.5	Additive
	Se-NPs + Cefipim	0.015625	0.078125	0.3125	Synergetic
	Se-NPS + Ceftrixone	0.03125	0.15625	0.1875	Synergetic
*E. coli* E0157:H7	Ceftrixone + ZnO-NPs	0.5	2.5	2.50	Indifferently
	Cefipim + ZnO-NPs	0.125	0.625	2.53	Indifferently
	Penicillin + ZnO-NPs	0.25	1.25	1.25	Additive
	Se-NPs + Penicillin	0.125	2.5	1	Additive
	Se-NPs + Cefipim	0.125	0.625	0.625	Additive
	Se-NPS + Ceftrixone	0.125	1.25	0.75	Additive
*E. coli* strain ESBL2-1	Ceftrixone + ZnO-NPs	0.5	2.5	1.50	Additive
	Cefipim + ZnO-NPs	0.125	0.625	1.28	Additive
	Penicillin + ZnO-NPs	0.25	1.25	0.75	Additive
	Se-NPs + Penicillin	0.125	2.5	1	Additive
	Se-NPs + Cefipim	0.125	0.625	0.625	Additive
	Se-NPS + Ceftrixone	0.125	1.25	0.75	Additive

Table 2 showcases the antibacterial properties and the impact of combining Se-NPs and ZnO-NPs with Ceftriaxone, Benzathine Penicillin, and Cefipime. When antibiotics were used against each type of NP, antibiotics exhibited varying MIC values depending on the bacterial strain. However, the MIC of the antibiotics decreased by a minimum of four times. This suggests that the antibiotics’ resistance decreased when combined with each of the NPs, resulting in the suppression of bacterial growth. The checkerboard method utilized a sequential dilution of Se-NPs and ZnO-NPs, against antibiotics, to identify possible synergistic effects. The findings, presented in Table 2, display the FIC index values for these various combinations. The MIC of each combination experienced a substantial reduction. Specifically, the MIC of ceftriaxone, when paired with Se-NPs and ZnO-NPs against MRSA#2 strain L2-15ST13, MRSA#2 strain L2-15ST13, *Escherichia coli* standard strain E0157:H7 and *Escherichia coli* strain ESBL2-1, decreased by 8- to 32-folds, Likewise, the MIC of penicillin combined with Se-NPs and ZnO-NPs lowered 8–16-fold and finally the MIC of cefipime with the ZnO-NPs and Se-NPs lowered from 8–20-fold. The computed FIC values for each pairing demonstrate that the combination of the synthesized Se-NPs and ZnO-NPs with the antibiotic Cefepime exhibited a synergistic effect against the two MRSA strains. In contrast, its interaction with *E. coli* strains resulted in an additive effect. While the FIC of the combination of each of synthesize Se-NPs and ZnO-NPs with the drug Cefipime has a synergetic effect upon treatment with the strains of MRSA strains and also the Se-NPs with drug ceftraxon has a synergetic effect upon treatment with the MRSA#2 strain L2-15ST13 while the FIC of the mixture of each synthesizes Se-NPs and ZnO-NPs with the 3 drugs had an preservative effect upon action with MRSA and *E. coli* Strains. Our results agree with previous  studies [31, 44] that have deliberated the synergistic effects have the potential to improve the antimicrobial activity in combination with antibiotics. Furthermore, this combination also improved the capacity to reverse the antibiotic resistance [45], and those findings were supported by alternative study evaluating the impact of a combination of ZnO-NPs with several antibiotics [46].

### Assessment of the expression of genes related to antibiotic resistance

The variation in the manifestation of antimicrobial resistance characteristics in a gram-positive bacterium (MRSA DSMZ28766) and a gram-negative bacterium (*Escherichia coli* DSMZ923) is influenced by the presence of either the antimicrobial agent alone or NPs alone. This is compared to the response observed when both NPs and the antimicrobial agents are used in combination, with results standardized against 16sRNA expression. The findings of this study indicate that the antimicrobial resistance traits of MRSA DSMZ28766 and *E. coli* DSMZ5923 were expressed when exposed to antibiotics, Se-NPs, and ZnO-NPs individually. However, these resistance traits were suppressed when cured with a mixture of NPs and antibiotics (Table 3). This confirms the combined and enhanced effects resulting from the interaction of Se-NPs and ZnO-NPs with Benzathine penicillin, Ceftriaxone, and Cefipime. Previous reports showed the effect of NPs’ introduction on the demonstration of antibiotic-resistant qualities in bacterial strains [27]. To the best of our knowledge,  information about exploring the quality expression of antibiotic-resistant qualities in bacterial strains when uncovered to NPs is rare. Consider these key aspects to understand how NPs operate at the genetic level in combating antibiotic-resistant microorganisms. Analyzing changes in quality expression can give understanding into how NPs are associated with bacterial cells and influence their resistance instruments. This information is significant for creating viable techniques to resist antibiotic-resistant microscopic organisms and can lead to the improvement of novel medications and intercessions.

**Table 3:** Antibiotic gene expression of bacterial strains treated with the myco-synthesized Se-NPs, ZnO-NPs, three antibiotics, and the combination between them

Treatment	*E. coli* DSMZ5923			MRSA DSMZ28766			
	blaTEM	blaCMY	blaSHV	MecA	Blaz	PBP-1	PBP-4
Penicillin	4.86 ± 0.586	6.70 ± 0.211	3.51 ± 0.038	5.1 ± 0.224	12.25 ± 0.259	11.3 ± 0.141	3.69 ± 0.321
Ceftraxone	2.85 ± 0.39	6.08 ± 0.725	3.90 ± 0.590	3.13 ± 0.37	8.38 ± 0.961	4.07 ± 0.296	3.97 ± 0.759
Cefipime	4.46 ± 0.278	3.36 ± 0.576	2.59 ± 0.396	5.07 ± 0.711	2.36 ± 0.015	2.14 ± 0.668	2.99 ± 0.525
Ceftrixone + ZnO-NPs	0.01 ± 0.005	0.13 ± 0.010	0.11 ± 0.061	0	0.01 ± 0.006	0	0
Cefipim + ZnO-NPs	0.01 ± 0.005	0.15 ± 0.040	0.16 ± 0.068	0.04 ± 0.004	0.15 ± 0.033	0.15 ± 0.046	0.04 ± 0.022
Penicillin + ZnO-NPs	0.05 ± 0.034	0.26 ± 0.031	0.46 ± 0.049	0.55 ± 0.023	0.57 ± 0.031	0.21 ± 0.069	0.15 ± 0.058
Se-NPs + Penicillin	0.01 ± 0.001	0.39 ± 0.053	0.25 ± 0.084	0.52 ± 0.032	0.57 ± 0.005	0.31 ± 0.113	0.21 ± 0.05
Se-NPs + Cefipim	0.01 ± 0.005	0.12 ± 0.031	0.18 ± 0.012	0.03 ± 0.001	0.2 ± 0.025	0.01 ± 0.008	0.02 ± 0.003
Se-NPS + Ceftrixone	0.01 ± 0.002	0.06 ± 0.005	0.06 ± 0.030	0.43 ± 0.011	0.39 ± 0.061	0.22 ± 0.065	0.18 ± 0.086
ZnO-NPs	1.09 ± 0.227	1.62 ± 0.463	2.95 ± 0.690	0.53 ± 0.011	1.12 ± 0.287	0.84 ± 0.457	1.15 ± 0.136
Se-NPs	0.74 ± 0.313	1.28 ± 0.708	3.49 ± 0.048	0.82 ± 0.167	1.23 ± 0.012	0.55 ± 0.164	0.37 ± 0.043
LSD	0.001	0.003	0.001	0.005	0.021	0.030	0.011

### Enhancement of the myco-synthesis efficiency using gamma irradiation

**Fig. 7:**
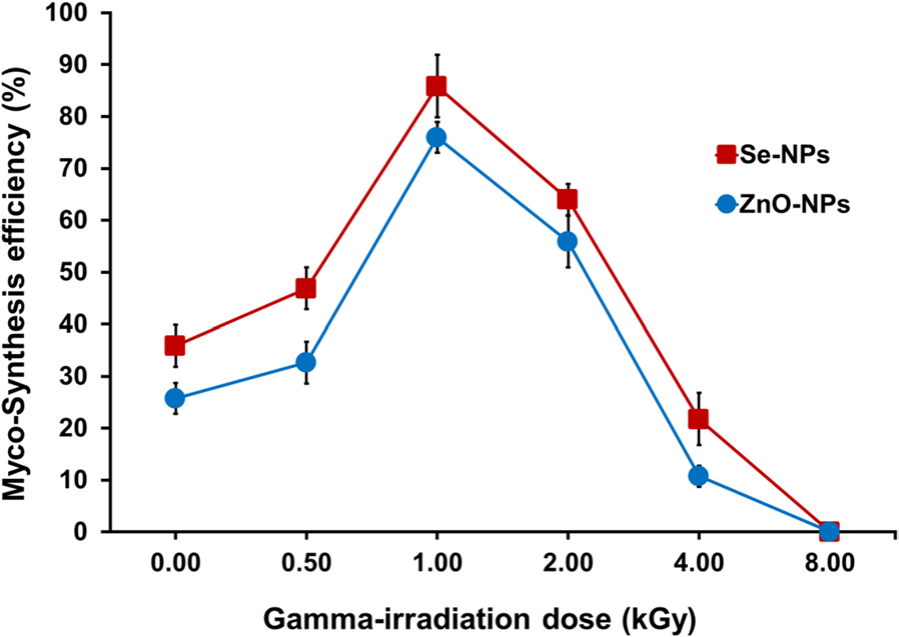
Effect of gamma irradiation on the synthesis efficiency of Se-NPs and ZnO-NPs by *Alternaria alternata* AUMC15177 fermented sugarcane bagasse extract

Recent studies in bionanotechnology have primarily focused on the fruitful fungal production of numerous nanomaterials for endless products [47]. In any case, the utilization of microbial strains for the improvement of a maintainable blend prepare of these nanomaterials has risen as a more current region of interest [48]. Thus, the myco-synthesis efficiency of Se-NPs and ZnO-NPs using extract of the fermented sugarcane bagasse from the *Alternaria alternata* AUMC15177 was followed after gamma irradiation at several doses. The obtained data (Fig. 7) indicated that 1000 Gy gamma rays enhanced the myco-synthesis effectiveness of Se-NPs and ZnO-NPs. At this dose, the highest efficiencies recording 85.87% (Se-NPs) and 75.98% (ZnO-NPs) was achieved. Compared to the control non-irradiated cultures, the improvement in the respective efficiencies was approximately threefold increase (Fig. 7). Multiple studies have explored the application of gamma irradiation at different exposure levels to enhance the production ofNPs. For instance, *A. terreus* has been utilized to synthesize metal oxide NPs [48], while *Monascus purpureus* [47] has facilitated the formation of Se-NPs and cobalt-ferrite NPs. Additionally, *Alternaria tenuissima* has been reported to generate ZnO-NPs [32], and *A. terreus* has also been employed in the biosynthesis of cobalt and copper NPs [31]. It is well established that when fungal cells are subjected to gamma radiation at particular levels [49–52], genetic mutations can be triggered [53–57], leading to an increased synthesis of secondary metabolites  [58–62]. Such metabolites are the key factors in the myco-reduction and synthesis process of Se-NPs and ZnO-NPs.

## Conclusion

Se-NPs and ZnO-NPs were effectively synthesized utilizing the endophyte *Alternaria alternata* AUMC15177. The myco-synthesized Se-NPs and ZnO-NPs showed a cytotoxic potential against the tested cell lines. Moreover, it exhibited notable antibacterial properties, as the resistance genes in the analyzed bacterial strains were suppressed when exposed to a blend mixture of  NPs and antibiotics. This highlights the enhanced and synergistic effects of these combinations. Finally, the synthesis efficiency of both NPs by the fungus was enhanced using gamma irradiation. The presented research suggests the fungal strain as a green production platform of Se-NPs and ZnO-NPs with promising bioactivities.

## Data Availability

No datasets were generated or analysed during the current study.
